# Simvastatin Enhances the Cytotoxic Effects of Doxorubicin in a Mammary Adenocarcinoma Cell Model by Involving Connexin 43

**DOI:** 10.1002/jbt.70214

**Published:** 2025-03-11

**Authors:** Roberta Vitale, Stefania Marzocco, Ada Popolo

**Affiliations:** ^1^ Department of Pharmacy University of Salerno Fisciano Salerno Italy

**Keywords:** chemoresistance, connexin 43, doxorubicin, simvastatin

## Abstract

Gap Junctions channels formed by Connexins (Cx) provide intercellular communication enabling the coordination of cell growth, differentiation, and metabolism, and their reduction has been shown in many tumor types. Expression levels of Cx43, the most extensively studied Gap Junctions forming protein, are reduced or completely absent in breast cancer cells, while their overexpression correlates with increased cellular permeability to anticancer agents and, consequently, reduced resistance to drug treatments. So, drug associations targeting Cx43 are being considered to overcome chemoresistance. Previous studies demonstrated that Simvastatin (Sim) interferes with Cx43 expression and localization, and chemo‐sensitizing effects of Sim in several tumor cell lines treated with antineoplastic chemotherapeutics have been shown. This study aimed to evaluate whether Sim cotreatment enhances Doxorubicin‐induced cytotoxicity by affecting Cx43 expression and/or phosphorylation, so MCF‐7 cells were treated with Sim (10 µM) for 4 h and then coexposed to Sim and Doxorubicin (1 µM) for 20 h. In Sim cotreated cells, increased membrane levels of Cx43 have been shown; moreover, decreased levels of Cx43 phosphorylated on Ser368 and Ser262 residues, involved in channel closure and disruption of cell–cell communication, have been demonstrated in these cells. In Sim cotreated cells increased Doxorubicin uptake and enhanced Doxorubicin‐induced cytotoxic effects have been demonstrated together with reduced migratory capacity. Our data support the notion that Sim cotreatment could be a possible strategy to overcome chemoresistance, since the observed increase in Cx43 membrane levels, and the concomitant reduction of Cx43 phosphorylation, could be responsible for increased sensitization of cells to Doxorubicin treatment.

## Graphical abstract

Simvastatin cotreatment increases Cx‐43 levels and reduces Cx43 phosphorylation on Ser262 and Ser368 in human breast cancer cells, thus enhancing Doxorubicin cellular accumulation and cytotoxic effects.

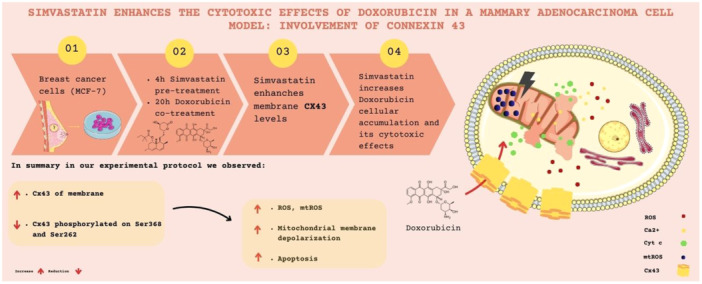

## 1 Introduction

Connexins (Cxs) are the main constituent of Gap Junctions (GJs), protein channels that provide the communication between adjacent cells [1]. In normal cells, the exchange of metabolites, ions, and small molecules through GJ is involved in cell growth, differentiation, and development [2]. In cancer cells, GJ intercellular communication (GJIC) is associated with the antitumor effects of therapeutic drugs since it enables the transmission of reactive oxygen species (ROS) and cytotoxic substances [3, 4]. The disruption of GJ networks resulting in the loss of these intercellular connections has been associated with multiple diseases, including cancer [5]. There are at least 21 connexin isoforms, named according to their molecular weight, but the most abundant and extensively studied is Cx43 [6]. Despite the role of Cx43 in cancer progression is some controversial, decreased expression of connexins and/or absence of GJIC have been associated with more aggressive phenotype in several types of cancer [7]. In breast cancer, Cx43 has been linked to the control of processes associated with breast cancer progression and metastasis, such as proliferation, invasion, migration, and apoptosis [8]. Indeed, several evidences indicate that decreased expression of Cx43 promote breast cancer cell migration [8, 9, 10, 11, 12], while Cx43 overexpression is associated with reduced invasion and metastatization, both in MDA‐MB‐231 breast cancer cells and in human breast cancer tissues [13]. Moreover, it has been demonstrated that a decrease in Cx43 expression results in a reduced chemotherapeutic drug diffusion between cells and a decreased cytotoxicity, while Cx43 overexpression increases the sensitivity of cancer cells to several chemotherapeutic drugs, such as Doxorubicin, 5‐FU, and Oxaliplatin in different type of cancers [14, 15, 16], thus reducing resistance to drug treatments [17, 18]. So, Cx43 could be an attractive target to overcome chemotherapy resistance that remains a major clinical concern in cancer treatment. To reduce the time and costs related to the development of a new drug, the repurposing drug approaches are receiving great attention, and Statins are being extensively studied in this context. Indeed, clinical data display that Statins, especially the lipophilic ones Simvastatin, Lovastatin, and Fluvastatin, can improve the effectiveness of chemotherapeutic drugs and the general condition of patients [19, 20]. Statins, commonly used as cholesterol‐lowering drugs, exhibit an important role in suppressing the growth of breast cancer cells [21, 22]. Simvastatin sensitizes MCF‐7 cells to Doxorubicin cytotoxic effects [23], and, overall, it has been demonstrated that Simvastatin induces a significant upregulation of Cx43, thus enhancing the effect of the chemotherapeutic drug Etoposide on cancer cells [24]. So, this study aimed to evaluate whether Simvastatin enhances the cytotoxic effects of Doxorubicin in MCF7 cells by affecting Cx43 expression and/or phosphorylation.

## 2 Materials and Methods

### 2.1 Reagents

Doxorubicin (Doxo, S‐5040420001) and Simvastatin (Sim, S6196) (both from Sigma, Milan, Italy) were used. Human breast cancer cell line (MCF‐7) was purchased from ATCC (Manassas, VA, USA) and cultured in 100‐mm Corning dishes containing Dulbecco's modified Eagle medium (DMEM; Gibco) with 10% fetal bovine serum (FBS), 25 U/mL penicillin, and 25 U/mL streptomycin in a humidified incubator at 37°C with 5% CO_2_.

### 2.2 Cell Treatment

MCF‐7 cells were plated at the necessary density for each of the different experimental assays and, after 24 h of adhesion, were treated with Sim (10 µM) for 4 h and then coexposed to Sim and Doxo (1 µM) for 20 h.

### 2.3 Morphological Analysis

For morphological analysis, MCF‐7 cells (5 × 10^4^ cells/well) were seeded into 24‐well tissue culture plates, allowed to reach 70%–80% confluence and treated as described above. Morphological changes were detected by light microscope magnification 5× (Zeiss, Axiovert 5).

### 2.4 MTT Assays

MCF‐7 cells (3.5 × 10^3^ cells/well into 96‐well plate) were treated as previously described. Cell viability was evaluated by means of 3‐(4,5‐dimethylthiazol‐2‐yl)‐2,5‐diphenyltetrazolium bromide (MTT) as previously reported [25], with minor revisions. After treatment, 25 µL of MTT (5 mg/mL) were added and cells were incubated for 3 h to allow the formation of formazan crystals, which were solubilized with 100 µL of DMSO. The optical density (OD) of each well was measured with a microplate spectrophotometer (Labsystems Multiskan EX) equipped with a 550 nm filter. MCF‐7 mortality was calculated as % of cell death = 100 − [(OD treated/OD control) × 100].

### 2.5 Flow Cytometry Analysis

MCF‐7 cells (4.5 × 10^5^ cells/well into 6‐well plate) were treated as reported above. To assess the membrane levels of Connexin 43 (Cx43), Connexin 43 phosphorylated on Ser368 (pCx43 Ser368) and Connexin 43 phosphorylated on Ser262 (pCx43 Ser262), cells were incubated with Fixing buffer (1% BSA, 1% formaldehyde in PBS) at 4°C for 20 min. Anti‐Cx43, anti pCx43 Ser368 or anti pCx43 Ser262 antibodies (all 1:250) were added along with the appropriate secondary antibody (anti‐rabbit FITC antibody; 1:250) for 30 min at 4°C. Instead, to assess intracellular levels of Cytochrome c (Cyt c), after incubation period with Fixing buffer, Fixing perm buffer (Fixing buffer containing 0.1% Triton X‐100) was added to allow membrane permeabilization. Anti Cyt c (1:250) and appropriate secondary antibody (anti‐mouse FITC antibody; 1:250) were added for 30 min at 4°C. Then cells were collected with Fixing buffer and fluorescence was evaluated by FACSscan using Cell Quest software. Results are shown as the percentage of positive cells.

### 2.6 Doxorubicin Cellular Accumulation

Intracellular uptake of Doxo was evaluated by spectrofluorimetric analysis as previously reported [26] with minor revisions. MCF‐7 cells (5 × 10^4^ cells/well into 24‐well plate) were treated as previously described, then three washes with 200 µL of cold phosphate‐buffered saline (PBS) were performed to remove the excess of Doxo. Fluorescence intensity was recorded at an excitation wavelength of 480 nm and an emission wavelength of 590 nm using a Spectofluorimeter (Perkin Elmer Enspire 2300 Multi‐mode Microplate reader).

### 2.7 Measurement of Intracellular Reactive Oxygen Species (ROS)

MCF‐7 cells (4 × 10^5^ cells/well into 6 well plate) were treated as described above. Cytosolic ROS levels were evaluated by means of H2DCF‐DA as previously reported [27]. Briefly, H2DCF‐DA (10 µM) was added for 15 min, then cells were collected with staining buffer (2% BSA, 0,1% Sodium Azide in PBS). Cell Fluorescence was evaluated by means of fluorescence‐activated cell sorting (FACSscan; Becton–Dickinson) and analyzed by Cell Quest software (version number 5.2.1). Results are shown as % of DCF positive cells.

### 2.8 Measurement of Mitochondrial Superoxide Formation

Mitochondrial superoxide formation was evaluated by means of MitoSOX Red, a probe that is readily oxidized by superoxide, but not by other ROS‐generating systems, and exhibits red fluorescence. MCF‐7 cells (4.5 × 10^5^ cells/well into 6‐well plates) were treated as previously described. MitoSOX Red (2.5 µM) was added for 15 min at 37°C, then the cells were harvested with Staining Buffer (2% BSA and 0.1% Sodium Azide in PBS). MitoSOX Red Cell Fluorescence was evaluated by means of FACSscan and analyzed by Cell Quest software (version number 5.2.1). Results are shown as % of MitoSOX positive cells.

### 2.9 Measurement of Intracellular Calcium Signaling

Intracellular calcium levels were evaluated by means of the fluorescent indicator Fura 2‐AM, the membrane‐permeant acetoxymethyl ester form of Fura 2. For these experiments, MCF‐7 cells (3 × 10^4^ cells/well into 12‐well plates) were treated as previously described. After treatment, cells were washed in PBS and resuspended in 1 mL of Hank's balanced salt solution (HBSS) containing 5 μM Fura 2‐AM for 45 min. To remove excess Fura 2‐AM, cells were washed with the same buffer, incubated in calcium‐free HBSS/0.5 mM EGTA buffer for 15 min to allow hydrolysis of Fura 2‐AM into its active‐dye form, Fura 2, and then transferred to the Spectrofluorimeter (Perkin‐Elmer LS‐55; Waltham, MA, USA). The mitochondrial calcium depletory, carbonyl cyanide p‐trifluoromethoxy‐pyhenylhydrazone (FCCP, 0.05 μM final concentration) and the calcium ionophore, Ionomycin (1 μM final concentration), were added into the cuvette in calcium‐free HBSS/0.5 mM EGTA buffer. Since the ratio of fluorescence intensity of 340/380 nm (F340/F380) is strictly related to intracellular free calcium [28, 29], the excitation wavelength was alternated between 340 and 380 nm, and emission fluorescence was recorded at 515 nm. Data were expressed as percentage of delta increase in fluorescence ratio (F340/F380 nm) induced by FCCP (0.05 mol/L) or Ionomycin (1 μmol/L) − basal fluorescence ratio (F340/F380 nm)/basal fluorescence ratio (F340/F380 nm).

### 2.10 Measurement of Mitochondrial Membrane Depolarization

The fluorescent dye tetramethylrhodamine methyl ester (TMRE) was used to evaluate mitochondrial permeability transition pore (mPTP) opening. TMRE, due to its positive charge, penetrates and accumulates in the mitochondria inversely proportional to the membrane potential. MCF‐7 cells (2 × 10^5^ cells/well into 12‐well plate) were treated as reported above. TMRE (5 nM) was added, then the plate was centrifuged at 1500 rpm for 5 min. Cells were collected with Staining buffer (0.1% Sodium Azide and 2% BSA in PBS). Cell fluorescence was evaluated using a fluorescence‐activated cell sorting and analyzed with Cell Quest software. Data are expressed as a percentage of TMRE low as previously reported [30].

### 2.11 Analysis of Apoptosis

Apoptosis was analyzed using propidium iodide (PI), a fluorochrome that is capable of binding cellular DNA content. MCF‐7 cells (4.5 × 10^5^ cells/well into 6‐well plate) were treated as described before and, as previously reported [31], a solution containing PI (50 µg/mL), 0.1% Triton X‐100 and 0.1% sodium citrate was added. The plate was then incubated at 4°C for 30 min in the dark and cell nuclei were analyzed by means of FACS using Cell Quest software. Data are expressed as a percentage of hypodiploid nuclei.

### 2.12 Scratch Assay

To determine the migratory potential of MCF‐7 cells, a scratch assay was performed. MCF‐7 cells (5 × 10^4^ cells/well into 24‐well plate) were plated, and scratch was created using 200 µL pipette tip, as previously described [32]. PBS was used to remove cells debris, fresh media was added and then cells were treated as described above. Migration of cells was observed at *t* = 0 and *t* = 24 h and scratch closure was captured by light microscope magnification 5× (Zeiss, Axiovert 5). Scratch area was measured with Image J software.

### 2.13 Statistical Analysis

Statistical analysis was performed with GraphPad Prism8 (GraphPad Software Inc. San Diego, CA). Data are reported as mean ± S.E.M. for at least three independent experiments, each performed in duplicate. Statistical analysis was performed by one‐way analysis of variance (ANOVA), followed by Bonferroni's posttest. A *p* value lower than 0.05 was considered statistically significant.

## 3 Results

### 3.1 Effect of Simvastatin Cotreatment on Doxorubicin‐Induced Cell Death

**Figure 1:**
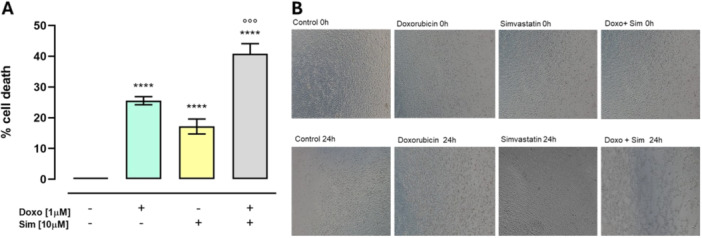
Effects of Simvastatin cotreatment on Doxorubicin‐induced cytotoxicity and morphological changes in MCF‐7 cells. Cellular death was assessed by MTT assay. Cell viability was calculated as % of dead cells = 100 − ([OD treated/OD control] × 100) (A). Light microscope was used to evaluate morphological changes in our experimental conditions (B). Data were analyzed by variance test analysis and multiple comparisons were made by Bonferroni's test. ****p < 0.001 versus control cells; °°°*p* < 0.005 versus Doxo‐treated cells.

MTT assay was performed to verify that, under our experimental conditions, Doxo was able to induce cytotoxic effects without reaching excessively high levels that would not allow the effects of Sim cotreatment to be detected. Our data showed that both Doxo and Sim alone significantly increased (*p* < 0.001) cell death compared with control cells. Sim cotreatment significantly (*p* < 0.005) increased the cytotoxic effects of Doxo (Figure 1A). As reported in Figure 1B, morphological changes were also evident in Doxo‐treated cells, and these were more prominent in Sim cotreated cells. Indeed, Sim cotreated cells showed considerable structural alterations due to shrinkage, rounding, and cell detachment compared with control cells and with Doxo‐treated cells.

### 3.2 Effect of Simvastatin Cotreatment on Cx43 and pCx43 Membrane Levels

**Figure 2:**
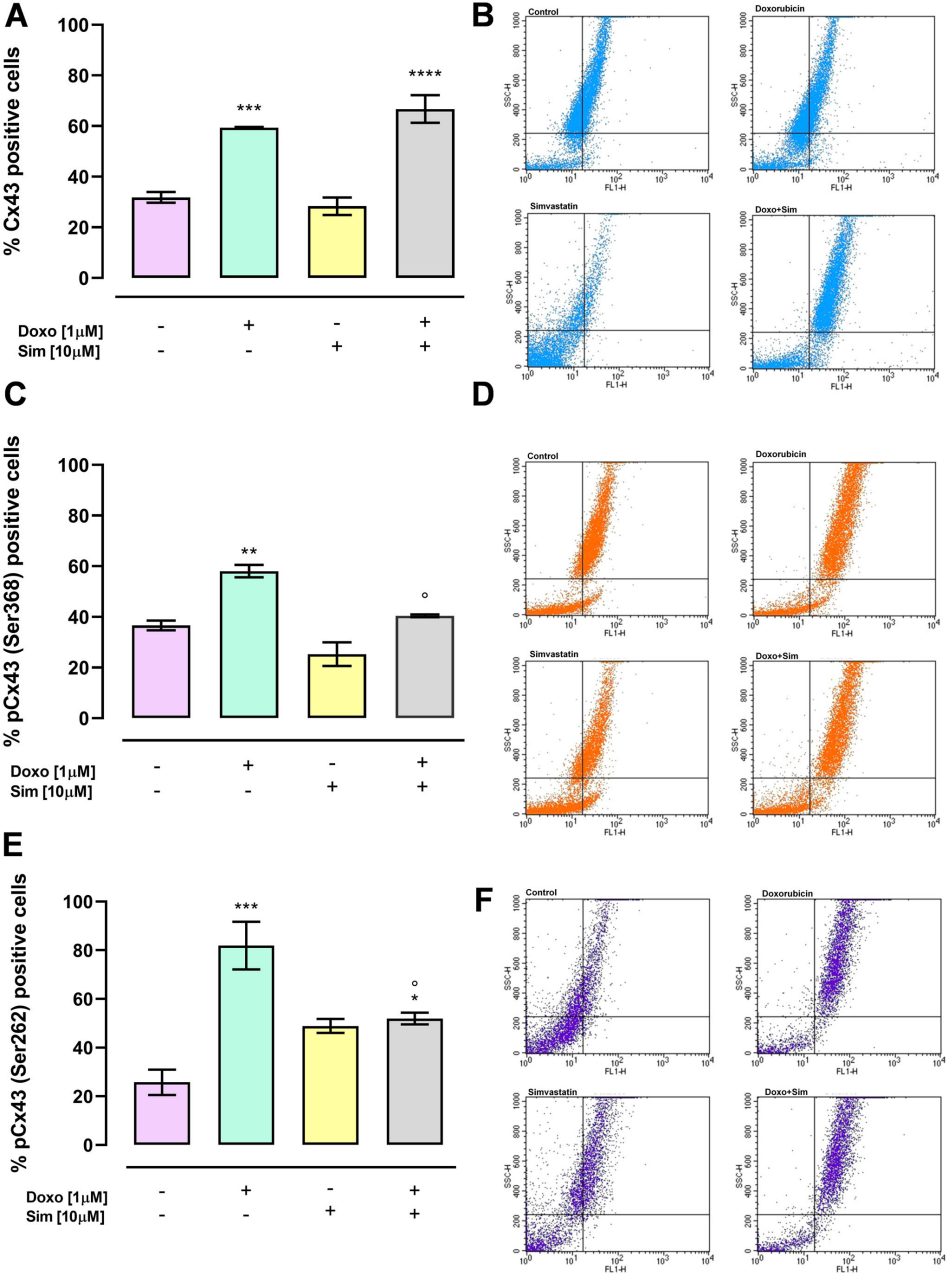
Simvastatin cotreatment alters Connexin 43 and Connexin 43 phosphorylated on Ser368 and on Ser262 levels. Membrane levels of Cx43 and pCx43 were detected by flow cytometry analysis in nonpermeabilized conditions. Results are reported as mean ± S.E.M. of percentage of Cx43 and pCx43‐positive cells from at least three independent experiments, each performed in duplicate (A, C, E). Representative histograms for the flow cytometry analysis are reported in (B, D, F). Data were analyzed by variance test analysis and multiple comparisons were made by Bonferroni's test. *p < 0.05, **p < 0.01, ***p < 0.005, ****p < 0.001 versus control cells; °*p* < 0.05 versus Doxo‐treated cells.

Previous studies reported that GJIC is lost during breast malignancy due to loss of Cx43 in the plasma membrane [33]. To test the hypothesis that Sim cotreatment affects Cx43 membrane levels, flow cytofluorimetric analysis in nonpermeabilzed conditions was performed. As reported in Figure 2A, Doxo‐treatment significantly (*p* < 0.005) increased Cx43 membrane levels compared to control cells. In Sim and Doxo cotreated cells a more significant (*p* < 0.001) increase in Cx43 membrane levels was observed compared with control cells even though no significant differences compared to Doxo‐treated cells were observed. Since phosphorylation of Cx43 plays a key role in regulating the trafficking, assembly, permeability, and disassembly of GJ [34], membrane levels of Cx43 phosphorylated on Ser368 and on Ser262 have also been evaluated. Doxo‐treatment induced a significant (*p* < 0.01) increase in membrane levels of Cx43 phosphorylated on Ser368 compared to control cells, but Sim cotreatment significantly (*p* < 0.05) reduced Doxo‐induced effects (Figure 2C). Also, membrane levels of Cx43 phosphorylated on Ser262 were significantly (*p* < 0.005) increased in Doxo‐treated cells while Sim cotreatment significantly (*p* < 0.05) reduced Doxo‐induced effects (Figure 2E).

### 3.3 Effect of Simvastatin on Doxorubicin Cellular Uptake

**Figure 3:**
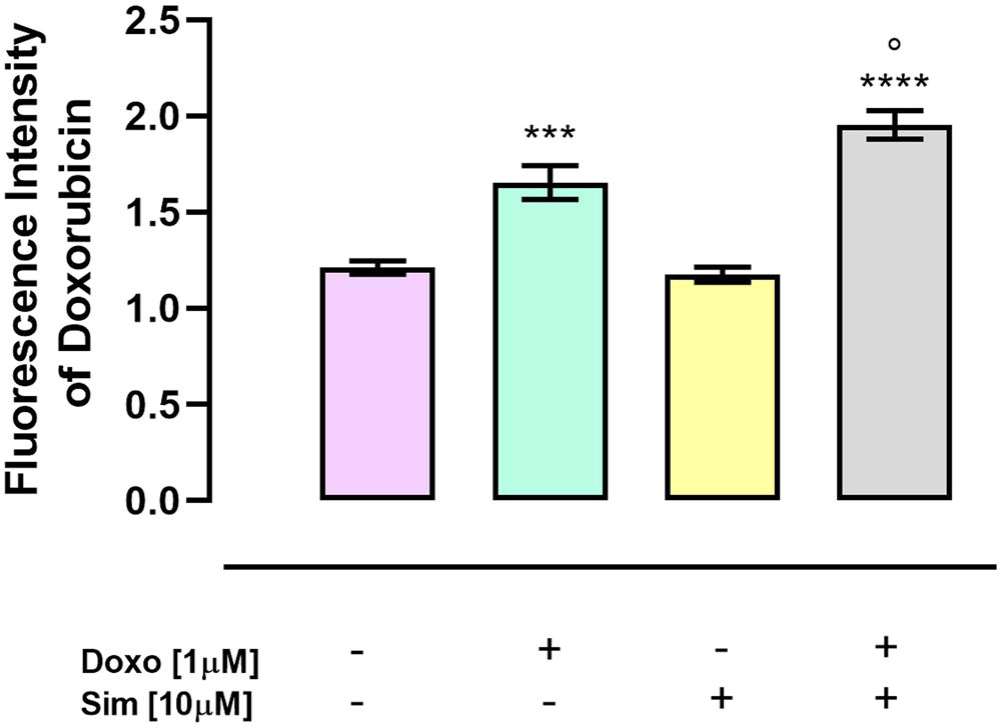
Simvastatin cotreatment increased Doxorubicin intracellular accumulation. Doxorubicin uptake was performed by spectrofluorimetric analysis. Results are reported as mean ± S.E.M. Fluorescence intensity of Doxorubicin from at least three independent experiments, each performed in duplicate. Data were analyzed by variance test analysis and multiple comparisons were made by Bonferroni's test. ***p < 0.005, ****p < 0.001 versus control cells; °*p* < 0.05 versus Doxo‐treated cells.

It has been shown that cells overexpressing Cx43 are more permeable to chemotherapeutic drugs than cells with low Cx43 expression [15, 35]. To evaluate Doxo intracellular uptake in our experimental conditions, spectrofluorimetric analysis was performed and our data showed that Sim cotreatment significantly (*p* < 0.05) enhanced Doxo intracellular uptake (Figure 3).

### 3.4 Effect of Simvastatin Cotreatment on Doxorubicin‐Induced Cytotoxic Effects

**Figure 4:**
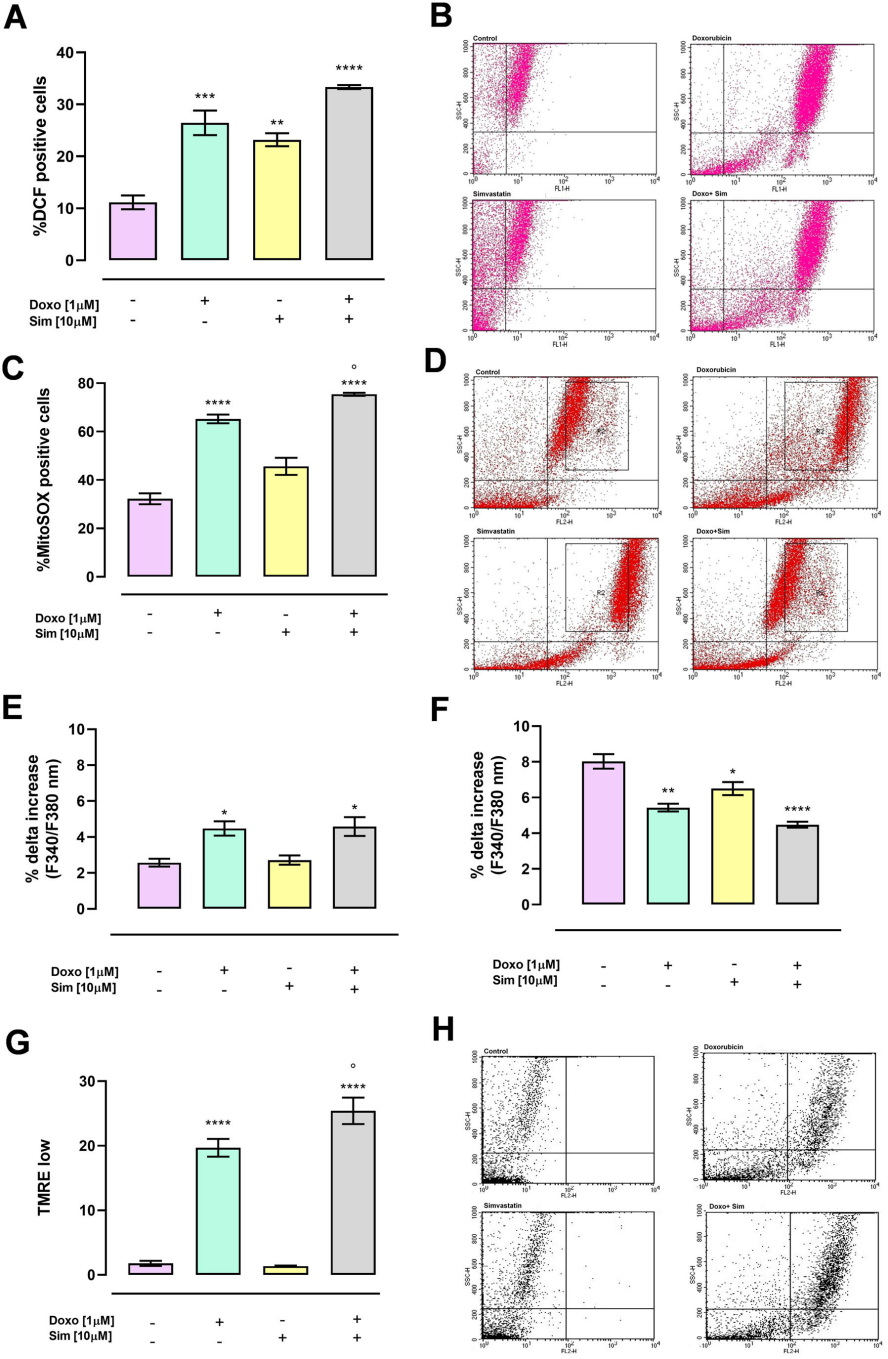
Simvastatin cotreatment enhances oxidative stress. Cytosolic and mitochondrial ROS production was evaluated by means of the fluorescent probe 2′,7′‐dichlorofluorescein diacetate (H2DCF‐DA) and MitoSOX Red, respectively, by flow cytometry analysis. Results are reported as mean ± S.E.M. of DCF‐positive cells percentage or Mito‐SOX Red‐positive cells (A and C) of at least three independent experiments, each performed in duplicate. FCCP (50 nM) in calcium‐free medium was used to evaluate mitochondrial calcium levels and Ionomycin (1 μM) in calcium‐free medium was used to evaluate intracellular calcium levels (E and F). Results are reported as mean ± S.E.M. of percentage of delta increase in FURA2 ratio fluorescence (340/380 nm) from at least three independent experiments, each performed in duplicate. The mitochondrial membrane potential was evaluated by flow cytometry analysis with tetramethylrhodamine ethyl ester (TMRE), a cationic dye that gives a strong fluorescence signal. Results are expressed as mean ± SEM of fluorescence intensity of at least three independent experiments each performed in duplicate (G). Representative histograms for the flow cytometry analysis are reported in (B, D, H). Data were analyzed by variance test analysis and multiple comparisons were made by Bonferroni's test*.* *p < 0.05, **p < 0.01, ***p < 0.005, ****p < 0.001 versus control cells; °*p* < 0.05 versus Doxo‐treated cells.

Doxo exerts its cytotoxic effects by enhancing intracellular ROS levels, dysregulating calcium homeostasis, and then affecting mitochondrial membrane potential, thus inducing apoptosis [36]. Increasing evidence suggested that the antineoplastic agents' cytotoxicity can be enhanced by modulating GJIC [37]. Here we evaluated if Cx43 increased levels affect Doxo sensitiveness in MCF‐7 cells and our data showed that Sim cotreatment significantly increased all Doxo‐induced cytotoxic effects. Indeed, data obtained by means of the fluorescent indicator DHCF showed that both Doxo and Sim alone significantly (*p* < 0.005 and *p* < 0.01, respectively) increased cytosolic ROS production compared with control cells, but in Sim and Doxo cotreated cells cytosolic ROS production was more significantly (*p* < 0.001) higher than control cells (Figure 4A). Moreover, cytofluorimetric analysis performed by means of the fluorescent indicator MitoSOX Red showed that Sim cotreatment significantly (*p* < 0.05) also increased Doxo‐induced mitochondrial ROS overproduction (Figure 4C).

Intracellular calcium content was evaluated by means of Fura 2‐AM, and our results showed that Doxo, both alone and in association with Sim, significantly (*p* < 0.05) increased the percentage of delta increase in calcium levels induced by the mitochondrial calcium depletory FCCP, indicating higher levels of calcium stored in mitochondria compared to control cells (Figure 4E). Regarding cytosolic calcium content, evaluated by means of Ionomycin administration, our data showed that both in Doxo and in Sim‐treated cells the percentage of delta increase was significantly (*p* < 0.01 and *p* < 0.05, respectively) lower than that in control cells. In Sim and Doxo cotreated cells, the percentage of delta increase in these cells were more significantly (*p* < 0.001) lower than control cells, indicating an impairment in intracellular calcium storage (Figure 4F). Data obtained by means of TMRE showed that Doxo induced a significant (*p* < 0.001) increase in mitochondrial membrane depolarization compared to control cells, and Sim cotreatment significantly (*p* < 0.05) increased Doxo‐induced mitochondrial membrane depolarization (Figure 4G).

**Figure 5:**
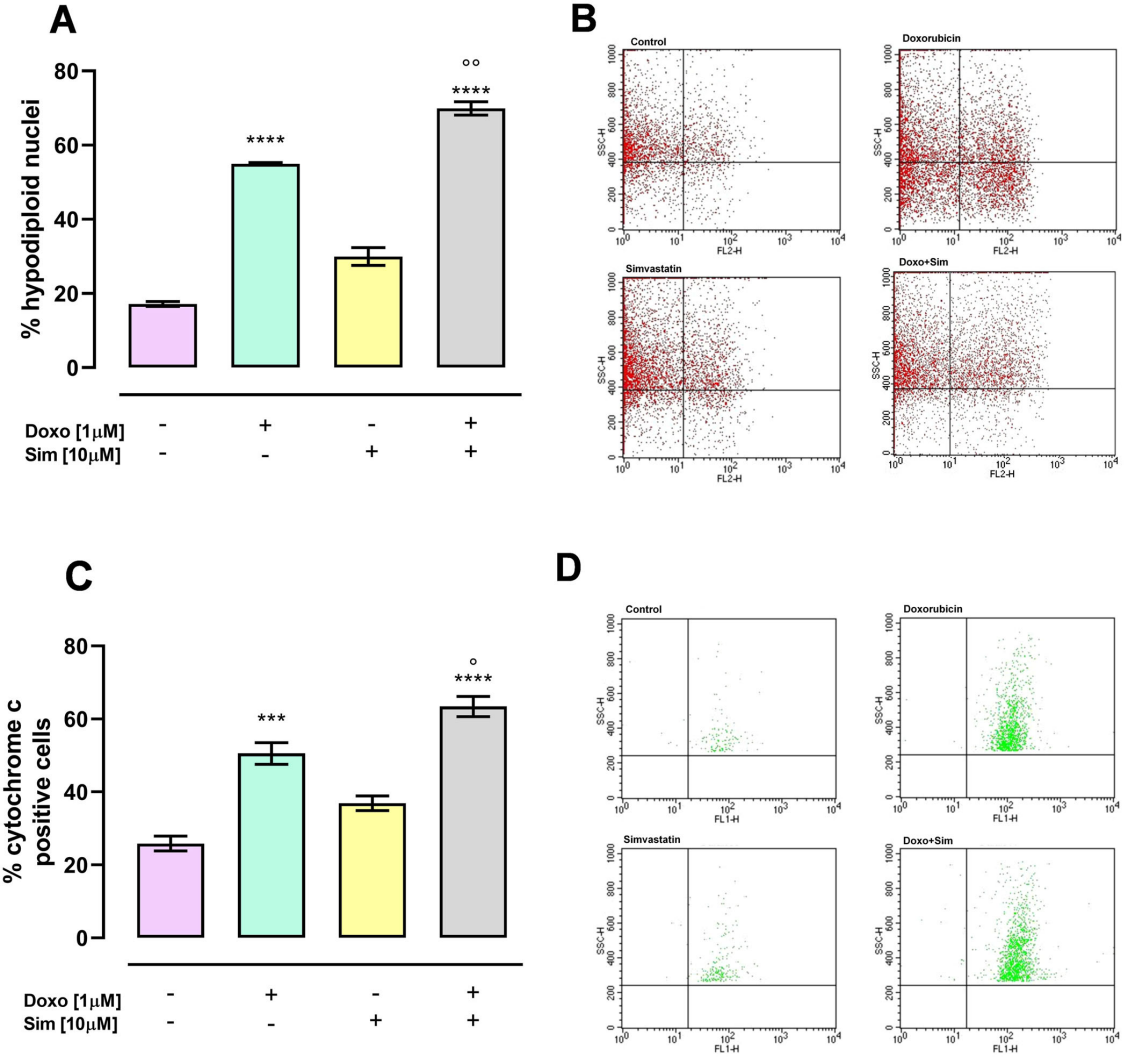
Simvastatin cotreatment enhances Doxorubicin‐induced apoptosis. MCF‐7 were stained by propidium iodide and fluorescence of individual nuclei was measured by flow cytometry. Results are reported as mean ± S.E.M. of % of hypodiploid nuclei from at least three independent experiments, each performed in duplicate (A). Flow cytometry analysis was used to evaluate cytosolic cytochrome c level. Results are reported as mean ± S.E.M. of % of cytochrome c positive cells from at least three independent experiments, each performed in duplicate (C). Representative histograms for the flow cytometry analysis are reported in (B and D). Data were analyzed by variance test analysis and multiple comparisons were made by Bonferroni's test*.* ***p < 0.005, ****p < 0.001 versus control cells; °*p* < 0.05, °°*p* < 0.01 versus Doxo‐treated cells.

Evaluation of the percentage of apoptotic cells, carried out using PI, showed that Doxo‐treatment significantly (*p* < 0.001) increased the percentage of hypodiploid nuclei compared with control cells and Sim cotreatment significantly (*p* < 0.01) increased Doxo‐induced apoptotic effcts (Figure 5A). The induction of the apoptotic pathway was confirmed by the evaluation of cytosolic content of Cyt c analyzed by cytofluorimetric analysis under permeabilizing conditions to allow the passage of antibodies at the cytoplasmic level. A significant increase (*p* < 0.005) in Cyt c release was observed in Doxo‐treated compared with control cells, and Sim cotreatment significantly increased (*p* < 0.05) Doxo‐induced Cyt c release (Figure 5C).

### 3.5 Effect of Simvastatin Cotreatment on Migratory Potential of MCF‐7 Cells

**Figure 6:**
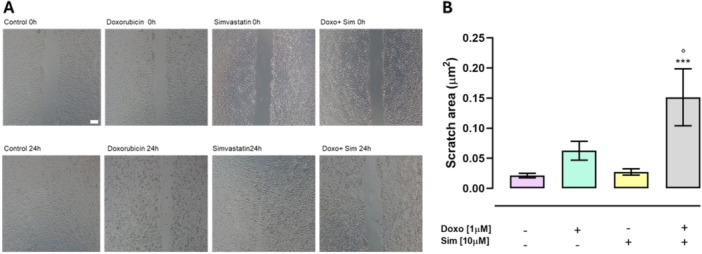
Simvastatin cotreatment reduces migration in MCF‐7 cells. Cell migration was assessed by Scratch assay. Scratch was captured after 24 h using a light microscope (scale bar 50 μm) to evaluate the effects of pretreatment with Simvastatin on migratory potential of MCF‐7 cells (A). Scratch area (µm^2^) was calculated by means of Image J software. Data are reported as mean ± S.E.M. of wound area (µm^2^) of three independent experiments, each performed in duplicate. Data were analyzed by variance test analysis and multiple comparisons were made by Bonferroni's test. ***p < 0.005 versus control cells; °*p* < 0.05 versus Doxo‐treated cells (B).

Cx43 expression is inversely related to cancer cells invasive and migratory abilities [7, 38]. A wound healing assay was performed to evaluate the effects of Sim cotreatment on the migratory potential of MCF‐7 under our experimental conditions. Doxo treatment affects the migratory ability of MCF‐7, since an increase of wound area, even if not significant compared to control cells, was observed. Sim cotreatment strongly increases the antimigratory effects of Doxo, since the wound area (µm^2^) was significantly (*p* < 0.05) higher than Doxo‐treated cells (Figure 6A).

## 4 Discussion

Breast cancer is still today one of the leading causes of cancer deaths in women [39]. Although many treatments are available, drug resistance remains a still concern, and the discovery of new clinical strategies able to improve chemotherapeutic efficacy while reducing chemoresistance to available drugs is paramount. Several studies demonstrated that breast cancer cells are characterized by reduced levels of Cx43 [33, 40], a GJ‐forming protein widely involved both in intercellular communication and in cancer progression and metastasis [6]. Data from in vitro study demonstrated that reduced expression of Cx43 is associated with increased invasiveness and metastatic capacity of cancer cells [41] and a meta‐analysis of transcriptome confirmed an association between loss of Cx43 expression and a poor prognosis [42]. It has also been shown that loss of communication between cancer cells reduces the spread of death signals, known as the by‐stander effect, and is associated with chemoresistance [17]. As a result, Cx43 has become an attractive target in cancer treatment, and many studies have focused on finding drugs that can interfere with it to improve the effectiveness of chemotherapeutic drugs [43]. Since the search of novel drugs is difficult, time‐consuming, and expensive, much attention has recently been paid to drug repurposing, that involves the use of drugs for diseases other than those for which they were originally intended [44]. In the context of overcoming chemotherapeutic resistance, interesting data came from Simvastatin [19], a lipid‐lowering drug that has been shown to have multiple effects that are unrelated to its ability to lower cholesterol, and has been associated with reduced cancer development and cancer‐related mortality [45, 46]. Simvastatin has been shown to exert a significant cytotoxic/cytostatic effect on the cancer cells [47, 48, 49] and, above all, affects Cx43 expression in cancer cells [50].

Based on these observations, this study aimed to test the hypothesis that Simvastatin can enhance the cytotoxic activity of Doxo on breast cancer cells (MCF‐7) by affecting Cx43 levels.

Cx43‐mediated GJIC involves only membrane proteins [33], but there is growing evidence that Cx43 is frequently downregulated or expressed in the wrong location in tumors [51]. We investigated the effect of Sim cotreatment on Cx43 membrane levels and reported a significant increase in Cx43 membrane levels in these cells. Cx43 is a phosphoprotein with at least 20 different phosphorylation sites involved in the Cx43 lifecycle, dynamic changes in GJ plaques, and GJs internalization [34, 52], with PKC‐mediated phosphorylation at Ser262 and Ser368 responsible for channel closure [53]. As previous studies have shown that Sim inhibits PKC the phosphorylation (and hence activation) [50, 54], we assessed the levels of Cx43 phosphorylated on Ser262 and on Ser368 and observed that Sim cotreatment significantly reduced them.

It has been reported that Cx43 is involved in drug delivery to cancer cells [6] as it has a greater capacity to transport macromolecules than other connexin proteins [15]. In addition, some drug metabolites or death signals induced in target cancer have been shown to be transferred via GJ to neighboring cells, significantly enhancing the efficacy of these chemotherapeutic agents [24]. Consistent with these data, we reported an increased intracellular accumulation of Doxo in Sim cotreated cells, which correlates with increased Doxo‐mediated cytotoxic effects. In fact, although both Sim and Doxo alone exert cytotoxic effects, increase oxidative stress, and affect cytosolic calcium homeostasis, more pronounced effects on mitochondrial ROS production and mitochondrial membrane depolarization with consequent induction of apoptosis were observed in Sim cotreated cells. Based on these results, we hypothesized that Simvastatin cotreatment would facilitate intercellular communication and cell death spread, by affecting both Cx43 levels and its phosphorylation status.

Besides its role in GJ formation, several studies showed that Cx43 is also involved in other noncanonical functions and highlighted its importance in regulating cellular functions including cancer cell migration [17, 41, 55]. It has been shown that a decreased expression of Cx43 promotes the metastasis of cancer cells [7, 38], since it is arguable that lack of connexins' connection induces tumor cells detachment from microenvironment and migration [40]. Our results from scratch assay demonstrated that Sim cotreated cells, which showed increased membrane levels of Cx43, exhibited reduced cell migration ability, thus supporting the correlation between Cx43 and migratory ability of cancer cells.

In conclusion, the data described in this article support the hypothesis that Simvastatin can be used as an adjuvant treatment in addition to Doxorubicin. Although further in vivo studies are needed to support our hypothesis, our results may have implications for the understanding of the central role of Cx43 in the development and treatment of cancer. Given the central role of Cx43 in intercellular death signals spreading and drug delivery, the discovery of drugs capable of acting on this target would overcome the problem of chemoresistance and, if these drugs are already in the market, would save a lot of time and money.

## Author Contributions

**Roberta Vitale:** writing – original draft, methodology, formal analysis, data curation. **Stefania Marzocco:** writing – review and editing, supervision. **Ada Popolo:** writing – review and editing, writing – original draft, funding acquisition, formal analysis, data curation, conceptualization.

## Data Availability

The data that support the findings of this study are available from the corresponding author upon reasonable request.
